# Effects of vegetation spatial pattern on erosion and sediment particle sorting in the loess convex hillslope

**DOI:** 10.1038/s41598-022-17975-6

**Published:** 2022-08-19

**Authors:** Yuanyi Su, Yang Zhang, Huanyuan Wang, Tingyu Zhang

**Affiliations:** 1 Key Laboratory of Degraded and Unused Land Consolidation Engineering, Ministry of Natural Resources, Xi’an, 710075 China; 2 Institute of Land Engineering and Technology, Shaanxi Provincial Land Engineering Construction Group Co., Ltd., Xi’an, 710075 China; 3 Shaanxi Provincial Land Engineering Construction Group Co., Ltd., Xi’an, 710075 China; 4 Shaanxi Provincial Land Consolidation Engineering Technology Research Center, Xi’an, 710075 China

**Keywords:** Ecology, Environmental sciences, Hydrology

## Abstract

To address the problem of serious soil erosion on the Loess Plateau, under the conditions of limited vegetation measures, the runoff erosion characteristics and erosion sediment sorting characteristics of vegetation at different positions on the upper slope of convex hillslopes are investigated, and the optimal vegetation spatial pattern is proposed according to the benefits of water storage and sediment reduction at different vegetation positions. The fluctuation degree of flow discharge per unit area of different vegetation spatial patterns is small, and the variation process of sediment discharge per unit area of each vegetation spatial pattern fluctuated sharply with the increase of runoff time. After planting vegetation on the slope, the total runoff yield and sediment yield were reduced. The runoff yield reduction benefit was 19.65% when the grass belt was 6 m away from the slope top; and the sediment yield reduction benefit was more than 70% when the grass belt was 2 m away from the slope top. Under the condition of hydraulic erosion on the slope covered with vegetation, the erosion particles are mainly fine particles, with high silt content and relatively small sand content. The farther the vegetation is arranged from the slope top, the more easily silt of size 0.002–0.05 mm is eroded. The higher effectiveness in terms of reductions of both runoff and sediment yields were obtained when the vegetation is planted in the proximity of the end of the length of the slope.

## Introduction

The area of the Loess Plateau in north-central China is characterized by thousands of gullies, complex terrain, low vegetation coverage, and is greatly affected by human activities, resulting in serious soil erosion, with an average annual soil loss of 5000 ~ 10,000 t/km^2^^1–5^. Increased soil erosion not only destroys the ecological environment, but also seriously hinders the sustainable development of the surrounding social economy^6–8^. Convex hillslopes are sections of slope located between gullies. Due to different slope types, the distribution of water in slope soil after rainfall infiltration varies, and the erosion and sediment yield characteristics of convex hillslopes are different from those of ordinary loess slopes. For example, Zhang et al.^9^ found that under the scouring condition of the convex hillslope, the slope velocity of the bare slope fluctuates considerably in space, while the erosion of the upper parts of both the upper and lower slopes are more serious. Therefore, the development of theory for the convex hillslope erosion process is not only the core issue of studying mechanisms of soil erosion in loess areas, but is also the key issue of controlling water and soil loss in these watersheds^10–12^.

In the study of soil erosion, there have been many previous studies investigating the regulation of vegetation on erosion and sediment yield. Several studies indicate that planting vegetation on slopes can effectively weaken runoff erosion power, improve soil erosion resistance, and inhibit water and soil loss^13–18^. In this way, reasonable vegetation spatial patterns can effectively improve soil properties, inhibit soil coarsening and reduce the loss of soil organic matter^19–22^. At the same time, some studies have shown that unreasonable vegetation spatial patterns can lead to more serious soil erosion^23,24^. Therefore, under a certain vegetation coverage, reasonable spatial patterns are the key to controlling water and soil loss. However, most of the above studies were conducted on straight slopes. Due to the particularity of erosion and sediment yield of convex hillslopes, it is necessary to strengthen the research on the spatial pattern of vegetation on convex hillslopes, along with its impact on erosion, sediment yield and soil properties.

As the particle size distribution characteristics of eroded sediment particles can well reflect the change process of erosion and the physical and chemical properties of the soil, studies of sediment particle size sorting have become an important index for the study of soil erosion processes^25–27^. For example, Slattery et al.^28^ found that at the beginning of erosion, the content of clay and silt in eroded sediment particles was high, and as erosion continued, the sediment particles become coarser with increased sand content, which stabilized over time. Wu et al.^29^ quantitatively studied the distribution characteristics of erosion sediment particles in the process of loess slope erosion, and determined that in the inter rill erosion stage, coarse particles decreased, fine particles increased and soil quality decreased. The particle size distribution characteristics of slope erosion sediment particles are affected by many factors, including soil texture, rainfall characteristics, runoff type, freeze–thaw effects and topographic characteristics^30–32^. The hydrodynamic process of soil erosion is changed after vegetation is planted on the slope. The ability of runoff to transport eroded sediment particles is reduced, resulting in changes in the particle size distribution characteristics of eroded sediment particles^33–37^.

Previous studies have mainly focused on the impact of vegetation on soil erosion on a single loess slope. However, complexity increases when the research object is the convex hillslope and the vegetation coverage is low. Therefore, through an indoor drainage and a scouring test, this study investigates the erosion reduction effect of vegetation and the sorting process of erosion sediment particles from the perspective of convex hillslopes, which seeks to further strengthen our understanding of the erosion process of loess slopes, as well as to optimize the reasonable pattern of vegetation. It has important scientific and practical significance for the management of convex slopes on the Loess Plateau.

## Materials and methods

### Test device and soil used in the experiment

**Figure 1:**
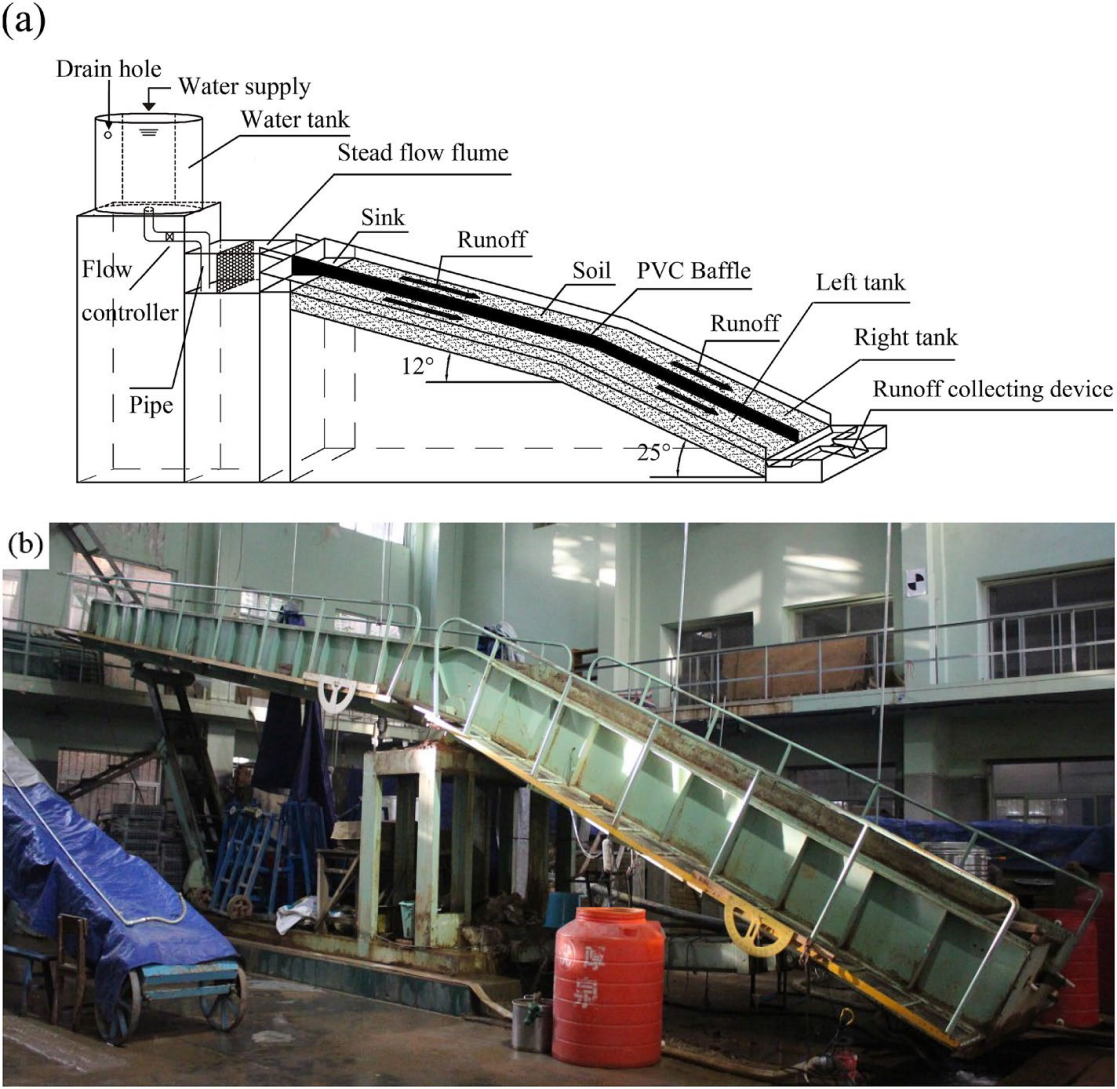
The generalization model of the convex hillslope. (**a**) The schematic diagram of convex hillslope model. (**b**) The photograph of convex hillslope model.

In this study, the convex hillslope in the hilly and gully region of the Loess Plateau in Northern Shaanxi, China was taken as the research object, and loess was used as the experimental soil. Determined by a Mastersizer 2000 (Malvern Instruments, UK) laser particle size analyzer, the soil particle composition was 12.93% clay, 82.55% silt and 4.52% sand. According to the soil classification standard of the United States Department of agriculture (USDA), the texture of the test soil was determined to be silty loam. According to the geomorphic characteristics of convex hillslopes of the Loess Plateau, the generalized physical model of the convex hillslope was established (Fig. 1a), and the generalized model test system was formed in combination with the laboratory infrastructure and the test design principles (Fig. 1b). The physical model was divided into two parts: the upper slope and the lower slope, with a width of 1 m, of which the upper slope was 8 m long with a slope of 12°, while the lower slope was 5 m long with a slope of 25°. Here, the horizontal projection area was 11.55 m^2^, and the length ratio of the upper slope to the lower slope was 1.6:1.0, which can effectively characterize the geomorphic characteristics of convex hillslopes in the hilly gully area of the Loess Plateau^38,39^. The soil tank for the slope ditch system of the generalized model was made of steel plate, where the middle of the soil tank was separated by PVC plate, which was divided into left tank and right tank to repeat the test. Two flumes with length 0.5 m, width 0.2 m and height of 0.2 m were placed on the top of the slope ditch system to maintain a consistent flow rate when entering the slope ditch system. The sediment and runoff during the experiment was picked up in a plastic bucket with a scale.

### Experimental design and methods

**Figure 2:**
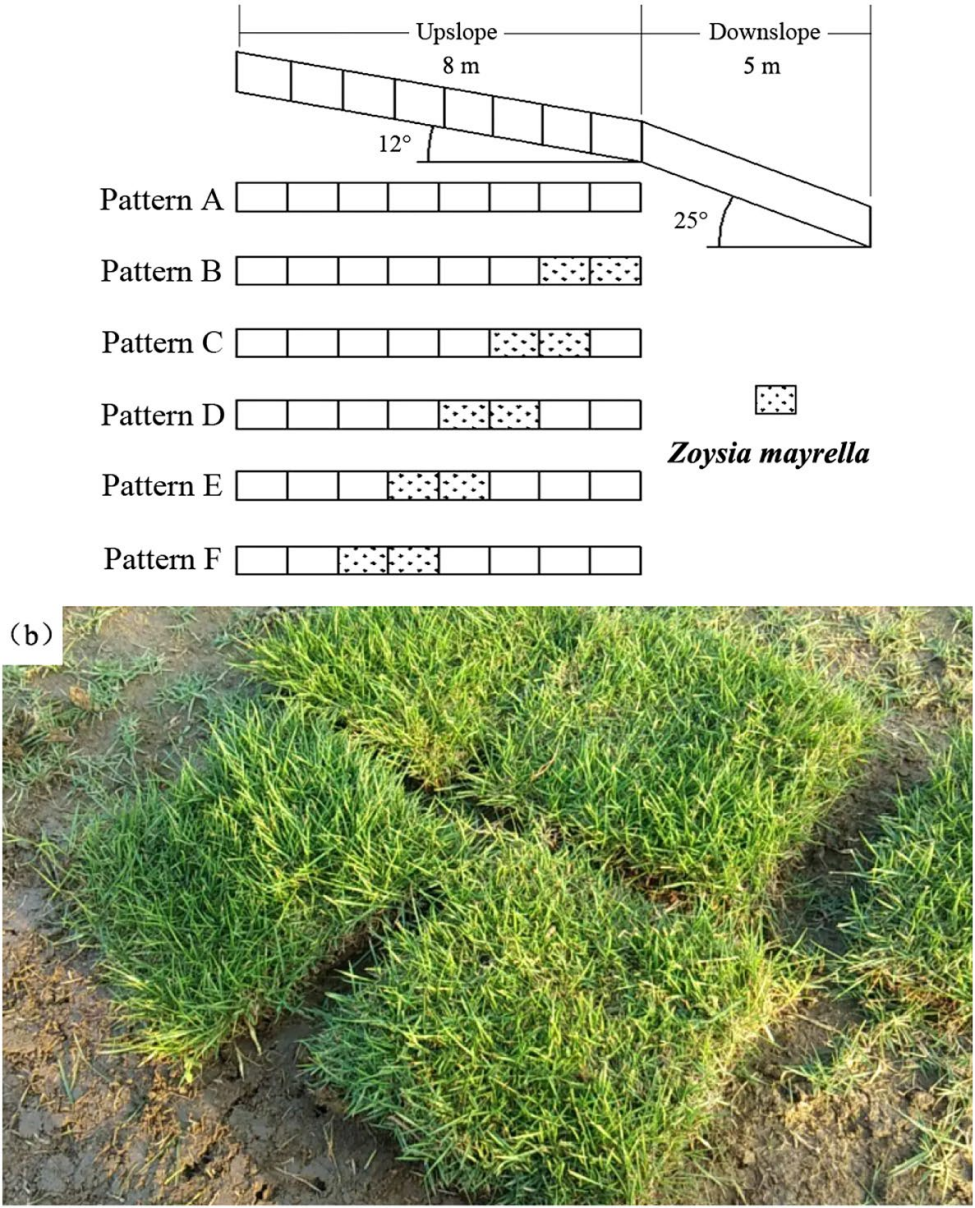
Schematic diagram of space position of vegetation on the upslope and *Zoysia matrella*. Pattern A (bare slope), patterns B–F (vegetation from 6 to 2 m from the slop top).

Based on the economic situation of the Loess Plateau, combined with the local drought and existing research on the benefits of vegetation water and soil conservation, the vegetation coverage for the water discharge scouring test was set as 25%^40^. The grass chosen for the experiment was *Zoysia matrella*, the grass belt size was 2 m (length) × 0.5 m (Width), with 20 cm root depth. In this study, the slope ditch system was divided into 13 sections, each of size 1 m (length) × 0.5 m (width). The spatial pattern of the grass belt in the convex hillslope is shown in Fig. 2. Pattern A is the bare slope, and the vegetation from 6 to 2 m from the slop top is Pattern B–F.

To ensure that the water permeability of the test soil was similar to the natural state, a 20 cm natural sand layer was paved at the bottom of the steel trough before the test. The test soil was loaded in layers, 5 cm for each layer, with a total of 4 layers. The soil was then compacted, and the soil bulk density was held at about 1.3 g/cm^3^. The soil was sprayed before the test for pre-wetting for an initial soil moisture content of about 20%. During filling, 10 cm thick of space was reserved at the position corresponding to the vegetation spatial pattern designed in the test. Two weeks before the test, the grass belt was transplanted to this part for natural growth, and the gap at the connection was filled and compacted to prevent the grass belt from sliding.

The test was conducted in the rain flood erosion Hall of Xi'an University of technology. The specific test design is shown in Table 1. Here, the rainfall data show that the heavy rain intensity of the loess area is about 90 mm/h, which is equivalent to a discharge flow of 16 L/min. The test was carried out after the flow was calibrated. Runoff and sediment samples were collected every minute after the beginning of runoff production at the collecting trough, and the runoff yield was measured. After standing for 24 h, the supernatant was poured out, the sediment sample was separated and placed in an oven at 105 °C for 8 h, and finally weighed to obtain the sediment yield. At the same time, the sediment samples after drying were collected and stored. After passing through a 2 mm sieve, the particle size of the sediment samples were measured using a Mastersizer 2000 laser particle size analyzer. The sediment samples were not subject to any dispersion treatment, and the measured data characterize the effective particle size distribution of sediment^37^. Each test had a duration of 30 min following the start of runoff production. Each group of tests was conducted three times, and finally the average value of the three tests was adopted.

**Table 1:** Designing table of vegetation spatial pattern for scouring experiment.

Vegetation spatial pattern	Scouring discharge (L min^−1^)	Position relative to slope top (m)	Vegetation coverage (%)	Runoff duration (min)
A	16	/	0	30
B	16	6	25	30
C	16	5	25	30
D	16	4	25	30
E	16	3	25	30
F	16	2	25	30

Pattern A (bare slope), patterns B–F (vegetation from 6 to 2 m from the slop top).

### Data calculation method

The calculation equations for flow discharge per unit area and sediment discharge per unit area are as follows.

1 $$ q^{\prime} = \frac{q}{T \cdot S} $$

2 $$ m^{\prime} = \frac{m}{T \cdot S} $$

where *q’* is flow discharge per unit area (L/(min m^2^)); *q* is the runoff yield (L); *m’* is sediment discharge per unit area (kg/(min m^2^)); *m* is the sediment yield (kg); *T* is runoff time (min); *S* is area of experiment slope, the size in this experiment is 0.4 m^2^.

Note: Runoff is the flow of water from rainfall down the surface of the earth under the action of gravity. Runoff yield refers to the amount of water passing through a certain water section in a certain period of time.

The relationship between cumulative runoff yield and cumulative sediment yield is as follows:

3 $$ M = aQ^{b} $$

where *M* is cumulative sediment yield (kg); *Q* is the cumulative runoff yield (L); *a* and *b* are correlation coefficients.

The particle sorting characteristics during erosion are expressed by the mean weight diameter (MWD)^41^, and its calculation formula is as follows:

4 $$ MWD = \frac{{\sum\nolimits_{1}^{i} {\chi_{i} \cdot \omega_{i} } }}{100} $$

where $\chi_{i}$ is the average value of grade *i* particles in mm; $\omega_{i}$ is the volume fraction of grade *i* particles expressed as a %. MWD is divided into three grades, which are classified according to the American agricultural standard, namely clay (< 0.002 mm), silt (0.002–0.05 mm) and sand (> 0.05 mm).

“Vegetation relative position index” expression is as follows:

5 $$ Z{ = }\frac{X}{Y} $$

where *Z* is the relative position index of vegetation; *X* is the distance from the center of the grass belt to the top of the upper slope (m); and *Y* is the distance from the center of the grass belt to the bottom of the downhill surface (m).

The calculation formula of runoff yield and sediment yield reduction benefits is as follows:

6 $$ R_{W} = (W_{A} - W_{\chi } )/W_{A} $$

7 $$ R_{S} = (S_{A} - S_{\chi } )/S_{A} $$

where *R*_*W*_ and *R*_*S*_ are the runoff yield and sediment yield reduction benefits under each vegetation spatial pattern (%); *W*_*A* (*χ*)_ is the total runoff yield under Pattern A and other vegetation spatial pattern (L); *S*_*A* (*χ*)_ is the total sediment yield under Pattern A and other vegetation spatial pattern (kg).

### Statistical analysis

All results are expressed as means ± standard deviations. Two way analysis of variance (ANOVAs) with a probability level of 0.05 was used to evaluate the impact of vegetation cover location on runoff yield, sediment yield and sediment particle sorting. Means were compared using Duncan's multiple range test for significant differences (*P* < 0.05). All statistical analyses were conducted using SPSS 21.0 (SPSS Inc., Chicago, USA).

### Ethical approval

The use of plants in the present study complies with international, national and/or institutional guidelines.

## Results

### Runoff process

**Figure 3:**
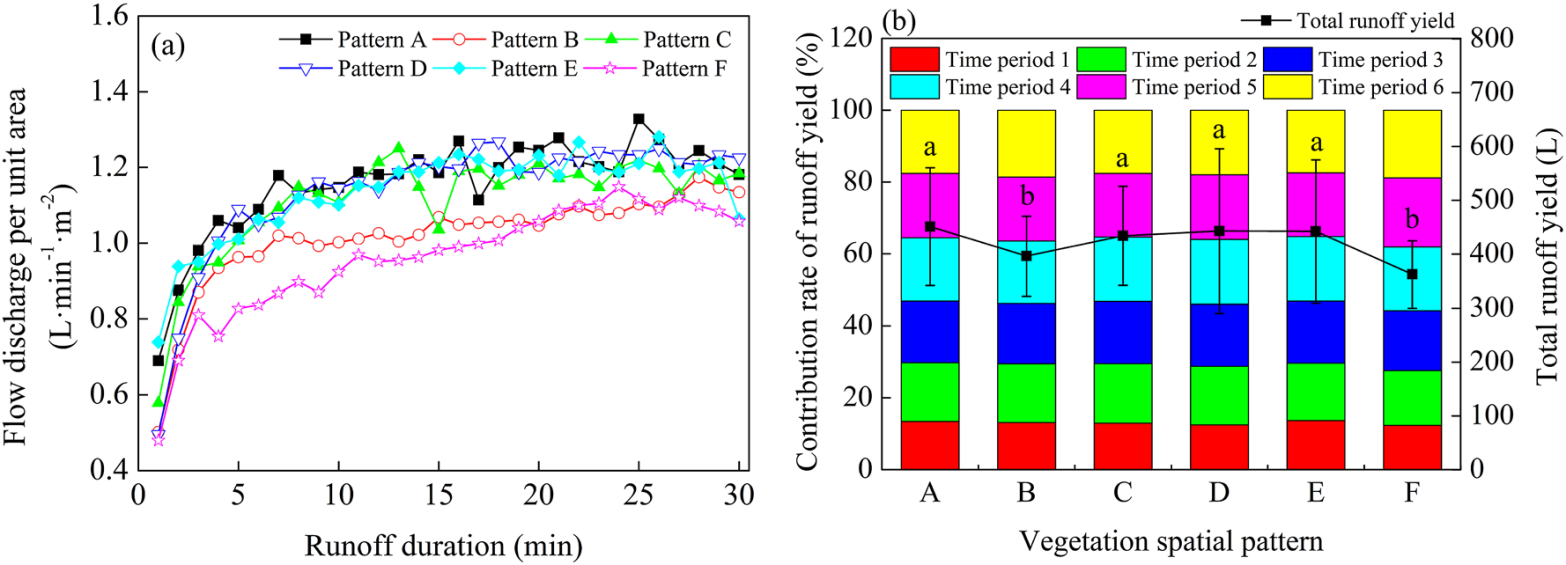
Runoff process and characteristics under different vegetation spatial patterns. (**a**) Flow discharge per unit area. (**b**) Total runoff yield. Different lowercase letters indicate significant differences between treatments in total runoff. Pattern A (bare slope), patterns B–F (vegetation from 6 to 2 m from the slop top).

In this study, the scouring test process was divided into six periods on average (the same to sediment process). The overall trend of flow discharge per unit area for each vegetation spatial pattern was roughly the same, where flow discharge per unit area first increased with runoff time and then gradually stabilized (Fig. 3, Table 2). The CV value of flow discharge per unit area for each vegetation spatial pattern was between 10.26 and 15.5%, and the fluctuation range of flow discharge per unit area was small (Table 2). Results from the ANOVA indicated that the flow discharge per unit area of Pattern A was significantly different from that of Pattern B and Pattern F (*P* < 0.05). Within 0–5 min, the flow discharge per unit area increased rapidly, with considerable fluctuation and was in an unstable state. As the test continued and soil water content increased, the soil infiltration rate decreased gradually, while the increasing rate of flow discharge per unit area decreased at 5–10 min, and stability was observed at 10–30 min (Fig. 3a). The proportion of runoff yield in period 1 of each vegetation spatial pattern to the total runoff yield was small, ranging from 12.33 to 13.63% (Fig. 3b). After the vegetation was arranged on the uphill surface, the total runoff yield decreased to varying degrees, indicating that the vegetation had played a certain role in water and soil conservation, among which the runoff yield reduction effect of Pattern F was the best, reaching a runoff yield reduction benefit of 19.65% (Table 2). Peak flow discharge per unit area under different vegetation spatial patterns following planting of the grass belt on the slope decreased to varying degrees compared with Pattern A, and the peak flow discharge per unit area was 0.86–0.96 times that of Pattern A.

**Table 2:** Characteristics value of runoff under different vegetation spatial pattern.

Vegetation spatial pattern	Fluctuation range (L min^−1^ m^−2^)	CV (%)	Average value (L min^−1^ m^−2^)	Peak flow discharge per unit area (L min^−1^ m^−2^)	*R*_*W*_ (%)
A	0.69 ~ 1.33	11.06	1.16 ± 0.28 a	1.33	/
B	0.50 ~ 1.17	12.88	1.02 ± 0.19 b	1.17	12.13
C	0.58 ~ 1.25	12.37	1.11 ± 0.24 a	1.25	3.79
D	0.49 ~ 1.27	14.48	1.14 ± 0.39 a	1.27	1.79
E	0.74 ~ 1.28	10.26	1.13 ± 0.34 a	1.28	1.95
F	0.48 ~ 1.15	15.50	0.96 ± 0.16 b	1.15	19.65

The data in the table are given as average ± SE, different lowercase letters within a column indicate significant difference between treatments. CV is coefficient of variation.

Pattern A (bare slope), patterns B–F (vegetation from 6 to 2 m from the slop top).

### Sediment process

**Figure 4:**
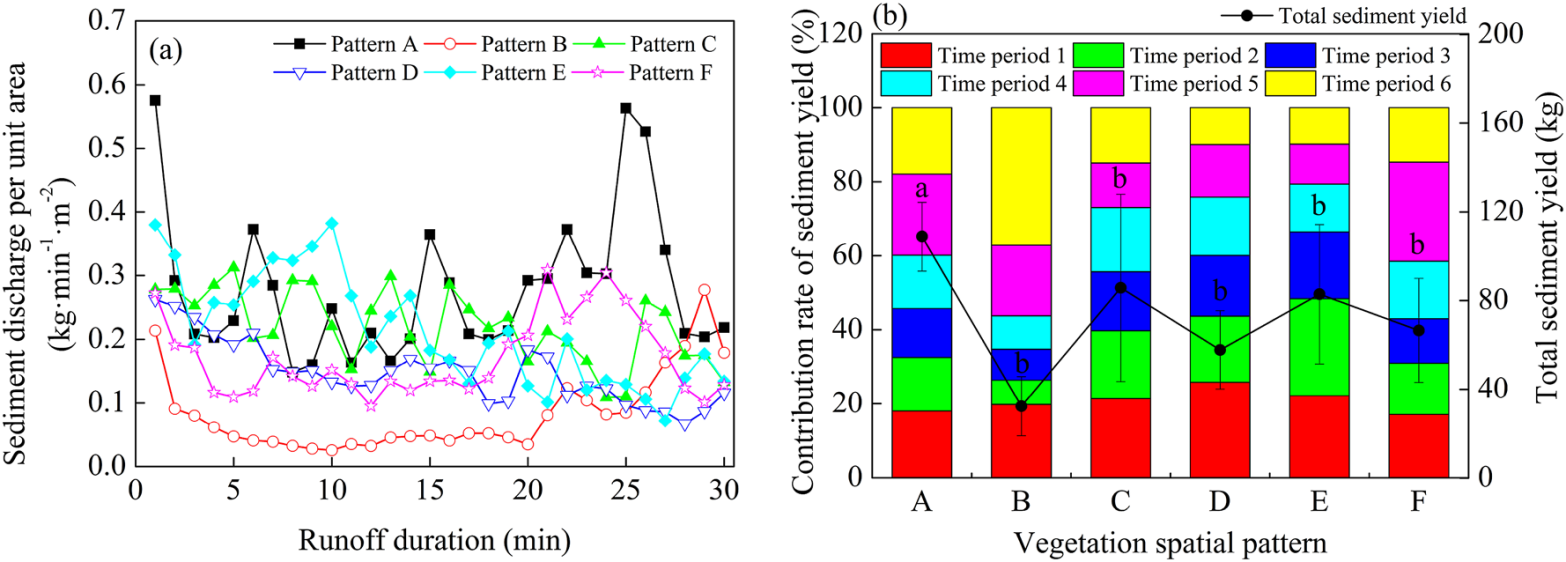
Sediment process and characteristics under different vegetation spatial patterns. (**a**) Sediment discharge per unit area. (**b**) Total sediment yield. Different lowercase letters indicate significant differences between treatments in total sediment yield. Pattern A (bare slope), patterns B–F (vegetation from 6 to 2 m from the slop top).

The sediment discharge per unit area fluctuated greatly with the increase of runoff production time, and the CV value was between 26.51 and 76.12% (Fig. 4, Table 3). At 0–5 min, the sediment discharge per unit area of different vegetation spatial patterns decreases gradually, and at 5–30 min, the sediment discharge per unit area fluctuated considerably (Fig. 4a). Results from the ANOVA test indicated that the sediment discharge per unit area of Pattern A was significantly different from that of the other patterns (*P* < 0.05), suggesting that the arrangement of the grass belt on the slope had a greater impact on the sediment process, and the impact of different vegetation spatial patterns on the sediment process was greater than that of the runoff process. Under the experimental conditions, the total sediment yield of Pattern B was the smallest, with a sediment yield reduction benefit as high as 70.22%, indicating that the grass belt arranged 6 m away from the slope top had a beneficial effect on direct sediment retention (Table 3). Although the total sediment yield is the smallest under Pattern B, the analysis of the contribution rate of sediment yield in different periods to the total sediment yield showed that, over time, the contribution rates of sediment yield in periods 5 and 6 reached 19.05% and 37.16%, respectively, indicating that the effect of vegetation sediment interception is gradually weakened with the extension of runoff time (Fig. 4b). The peak sediment discharge per unit area of different vegetation spatial patterns were 48.3% (Pattern B), 54.28% (Pattern C), 45.59% (Pattern D), 62.43% (Pattern E) and 53.74% (Pattern F) lower of Pattern A, respectively.

**Table 3:** Characteristics value of sediment under different vegetation spatial pattern.

Vegetation spatial pattern	Fluctuation range (kg min^−1^ m^−2^)	CV (%)	Average value (kg min^−1^ m^−2^)	Peak sediment discharge per unit area (kg min^−1^ m^−2^)	*R*_*S*_ (%)
A	0.15 ~ 0.58	40.66	0.28 ± 0.04 a	0.58	/
B	0.03 ~ 0.28	76.12	0.08 ± 0.03 b	0.28	70.22
C	0.11 ~ 0.31	26.51	0.22 ± 0.11 b	0.31	21.19
D	0.07 ~ 0.26	33.83	0.15 ± 0.05 b	0.26	46.88
E	0.07 ~ 0.38	41.69	0.21 ± 0.08 b	0.36	23.81
F	0.10 ~ 0.31	36.47	0.17 ± 0.06 b	0.31	38.84

The data in the table are given as average ± SE, where different lowercase letters within a column indicate a significant difference between treatments. CV is coefficient of variation.

Pattern A (bare slope), patterns B–F (vegetation from 6 to 2 m from the slop top).

### Cumulative runoff yield and cumulative sediment yield

**Figure 5:**
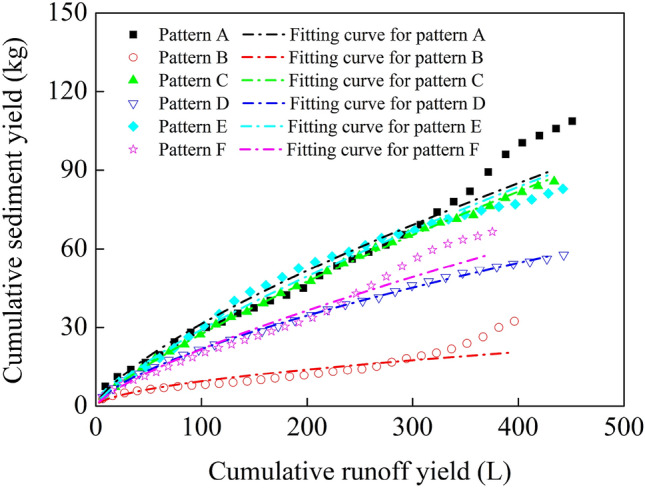
Relationship between cumulative runoff yield and cumulative sediment yield. Pattern A (bare slope), patterns B–F (vegetation from 6 to 2 m from the slop top).

Although the relationship between runoff yield and sediment yield in convex hillslopes is complex, previous studies have characterized the runoff yield and sediment yield relationship under different vegetation spatial patterns. In this study, the cumulative runoff yield and cumulative sediment yield under different vegetation spatial patterns were fitted and compared. Combined Fig. 5 and Table 4, it can be seen that the relationship between cumulative runoff yield and cumulative sediment yield was determined to be a power function. The fit coefficient of each curve reached more than 89%.

**Table 4:** Coefficients (*a*, *b*) and coefficient of determination (*R*^*2*^) of Eq. (3) under different vegetation spatial patterns.

Vegetation spatial pattern	*a*	*b*	Fitting coefficient *R*^*2*^ (%)
A	1.15	0.80	97.49
B	0.71	0.56	89.83
C	0.74	0.78	99.96
D	1.04	0.66	99.92
E	0.90	0.76	99.16
F	0.69	0.75	97.66

Pattern A (bare slope), patterns B–F (vegetation from 6 to 2 m from the slop top).

Figure 5 displays the relationship between cumulative runoff yield and cumulative sediment yield under different vegetation spatial patterns. Since no grass strips were laid on the slope of Pattern A, rills gradually formed on the slope at the later stage of the test process, and the sediment yield increased sharply, which caused a sudden change in the runoff yield–sediment yield relationship. For Pattern B and F, grass strips were laid at different locations on the slope, and the vegetation played a better role in runoff yield and sediment yield reduction, the runoff yield–sediment yield relationship also changed abruptly. Therefore, the fitting coefficients of Cumulative runoff yield and cumulative sediment yield for Pattern A, B and F were lower.

Equation (3) was fitted to the (*Q*, *M*) pairs related to cumulative runoff yield and sediment yield under different vegetation spatial patterns (Table 4). The ordered values of *a* are F < B < C < E < D < A, the ordered values of *b* are B < D < F < E < C < A. Through comparison, it is found that the water storage and sediment yield reduction benefits obtained with *a* and *b* as correlation coefficients are completely consistent with the actual water storage and sediment yield reduction benefits under different slope vegetation patterns. Therefore, the correlation between cumulative runoff yield and cumulative sediment yield of the convex hillslope under different slope vegetation patterns can be fitted by power function, and the correlation coefficients *a* and *b* can be used as indicators of water storage and sediment yield reduction benefits.

### Mean weight diameter (MWD)

**Figure 6:**
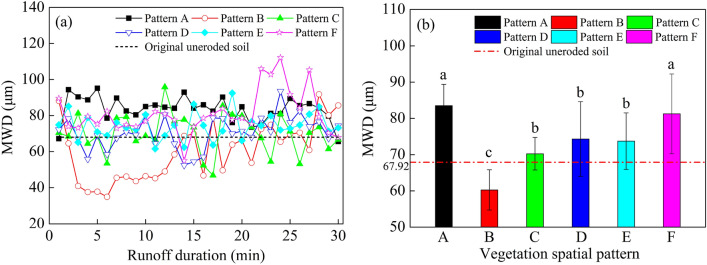
The average value of the mean weight diameter (MWD) and temporal variation of MWD of particles. Different lowercase letters represent significant difference at 0.05 level among different experiments. Pattern A (bare slope), patterns B–F (vegetation from 6 to 2 m from the slop top).

The MWD of Pattern A increased rapidly following the beginning of runoff production on the slope, which then decreased and remained stable, and finally gradually decreased and approached the MWD of the substrate at the end of runoff production (Fig. 6). The change law of MWD under Patterns C, D and E were relatively similar, fluctuating near the substrate throughout runoff production, and tending to the substrate at the end of runoff production. The MWD of Pattern B decreased rapidly at 0–6 min of runoff production, then increased and approached the MWD of the substrate at 6–15 min, and fluctuated violently and increased at 15–30 min. The MWD of Pattern F fluctuated slightly from 0 to 21 min, increased rapidly and then decreased after 21 min, and approached the MWD of the substrate at the end of runoff production.

Under the conditions of this experiment, the change in mean weight diameter (MWD) of the natural particles of eroded sediment was mainly influenced by the effects of vegetation spatial allocation along with slope runoff sorting characteristics. The ordered average values of MWD were: Pattern A > Pattern F > Pattern D > Pattern E > Pattern C > substrate > Pattern B. The MWD average value of Pattern A was the largest, which was 83.49 μm. The range in variation was 65.55–95.15 μm. The MWD average value of Pattern B was the smallest, which was 60.25 μm. The variation range for Pattern B was 34.92–91.85 μm. The results from the ANOVA indicated that there was no significant difference between the MWD of Pattern C, D and E, while the MWD of Pattern B was significantly different from that of the other patterns (*P* < 0.05), and the MWD of Pattern B was significantly smaller than that of the substrate (*P* < 0.05).

### Clay, silt and sand

**Figure 7:**
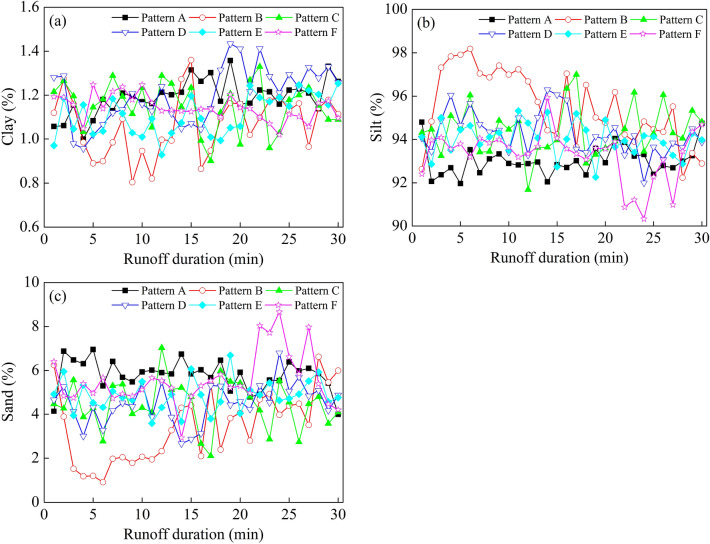
Variation of the effective particle size percentage of each grade of sediment with time under different vegetation spatial patterns. (**a**) Clay. (**b**) Silt. (**c**) Sand. Note: Pattern A (bare slope), patterns B–F (vegetation from 6 to 2 m from the slop top).

The clay content under each vegetation spatial pattern was very low, with no obvious change law with the extension of runoff generation time observed (Fig. 7a). Following runoff production on the slope, the content of silt for Pattern A decreased rapidly, then fluctuated up and down before becoming stable. After 25 min, the content of silt increased. Patterns C, D and E displayed similar changes over time. Silt content fluctuated from the beginning of runoff production and remained relatively stable until the end of the test. The content of Pattern B silt increased rapidly from 0 to 6 min, decreased from 6 to 15 min, and fluctuated with a decreasing trend from 15 to 30 min. The content of Pattern F silt fluctuated slightly from 0 to 21 min, decreased rapidly and then increased after 21 min (Fig. 7b). Under each vegetation spatial pattern, the change trend of sand content over time was opposite to that of silt (Fig. 7c).

To further reveal the variation law of sediment particles of each particle size, contents of different sediment particle sizes in the process of slope erosion under different vegetation spatial patterns were statistically analyzed (Table 5). The clay content under each vegetation spatial pattern was very small, accounting for only 1.07–1.20% of the total content. Silt content was higher, reaching more than 93%, of which the silt content for Pattern B was the largest, reaching 95.48%. As for sand, its content gradually decreased with the increasing distance from the grass belt layout position to the slope top, which may be related to the hydraulic conditions of the slope. Results from the ANOVA test showed that there was no significant difference in clay content under the different vegetation spatial patterns (*P* > 0.05), while there was significant difference in silt and sand content between Pattern B and the other patterns (*P* < 0.05).

**Table 5:** The average percentage of effective particle size of sediment under different vegetation spatial patterns.

Vegetation spatial pattern	Content (%)		
	Clay	Silt	Sand
A	1.19 ± 0.11 a	93.02 ± 8.31 a	5.79 ± 0.44 a
B	1.07 ± 0.12 a	95.48 ± 6.23 b	3.45 ± 0.30 b
C	1.15 ± 0.12 a	94.38 ± 9.15 a	4.47 ± 0.56 a
D	1.20 ± 0.06 a	94.29 ± 4.10 a	4.51 ± 0.62 a
E	1.11 ± 0.10 a	94.03 ± 6.20 a	4.86 ± 0.46 a
F	1.14 ± 0.13 a	93.30 ± 8.13 a	5.56 ± 0.67 a

The data in the table are given as average ± SE, different lowercase letters within a column indicate significant difference between treatments.

Pattern A (bare slope), patterns B–F (vegetation from 6 to 2 m from the slop top).

### Optimal allocation of vegetation pattern

**Figure 8:**
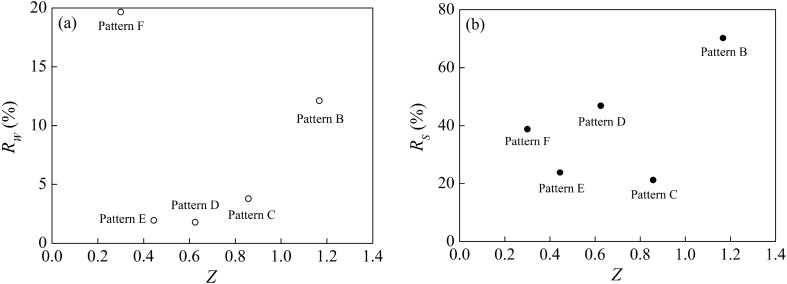
Regression results of relative positions of different vegetation and benefits of runoff yield and sediment yield reduction. (**a**) Runoff yield reduction function. (**b**) Sediment yield reduction function. Pattern A (bare slope), patterns B–F (vegetation from 6 to 2 m from the slop top).

According to the runoff yield and sediment yield reduction benefits under different vegetation spatial patterns, the layout position of the grass belt on the slope varied, and the runoff yield and sediment yield of the whole convex hillslope was different under each vegetation spatial pattern. A reasonable layout of the grass belt can play an effective role in soil and water conservation. Therefore, the spatial pattern of vegetation is particularly important in regulating the runoff yield and sediment yield of the convex hillslope. As mentioned above, Pattern F exhibited the greatest runoff yield reduction effect, that is, when the grass belt was 2 m away from the slope top, which had a beneficial runoff yield reduction effect. Pattern B displayed the best sediment reduction effect, that is, when the grass belt was 6 m away from the slope top, which generated a beneficial sediment reduction effect. However, the statement that the grass belt is 2 m or 6 m away from the slope top is absolute, and the index is a single value. Therefore, to avoid the disadvantage of using single index, the "vegetation relative position index" is used to determine the optimal area of the vegetation layout. According to the definition of the vegetation relative position index, *Z* ranges from 0.3 to 1.17. The relationship between the relative position parameters of vegetation and the benefits of runoff yield and sediment yield reduction are shown in Fig. 8. The image form of the fitting function of runoff yield and sediment yield reduction benefit and vegetation relative position index are roughly the same, that is, with the increase of vegetation relative position index, the runoff yield and sediment yield reduction benefit gradually decrease to the lowest value, and then increase (Fig. 8). When *Z* is 0.4–1.11, the runoff yield reduction benefit is less than 10%, meaning, when the vegetation layout position is relatively close to the middle of the slope, the runoff yield reduction effect of vegetation is relatively poor. With the grass belt layout position moving upward or downward, the runoff yield reduction benefit gradually increases. When *Z* is 0.3–1.03, the sediment yield reduction benefit is less than 50%, that is, when the vegetation layout position is relatively close to the middle of the slope, the sediment reduction effect of vegetation is relatively small. With the grass belt layout position moving upward or downward, the sediment yield reduction benefit increases gradually. Therefore, the range of *Z* values from 1.11 to 1.17 is defined as the optimal layout area for vegetation to reduce water and sediment. In this experiment, Pattern B is the only vegetation position that can assure both high runoff yield and sediment yield reductions.

## Discussion

### Effects of different vegetation spatial patterns on erosion process

The convex hillslope represents the most important part of the Loess Plateau. Its erosion mainly includes three processes: soil particle dispersion and stripping caused by rainfall and runoff, sediment transport and sediment deposition^42,43^. The process of soil erosion is complex, including influences from interactions of these interrelated processes. In contrast, vegetation measures are one of the three major factors related to soil and water conservation^44,45^. Vegetation can reduce runoff yield erosivity and improve soil erosion resistance along with the effect of soil consolidation and slope protection. Due to the extreme shortage of water resources in the Loess Plateau, vegetation restoration and reconstruction measures have become the most ideal choice for ecological environment construction^18,46,47^.

In this study, we showed that when the convex hillslope is planted with vegetation, the runoff yield and sediment yield are reduced to varying degrees, indicating that vegetation plays a certain role in water and soil conservation^48^. In terms of the water storage benefit of vegetation, each vegetation pattern within the test range performed at a low level, indicating that the effect of vegetation on reducing runoff yield is weak. In particular, the water storage benefit under the conditions of vegetation Patterns C, D and E were insufficient. In contrast, the benefit of sediment reduction under each vegetation pattern was significantly greater than that of water storage. These findings suggest that vegetation has a more beneficial effect on soil and water conservation through direct sediment interception, which is consistent with findings from previous studies^9,49–51^.

In comprehensively comparing the beneficial values of water storage and sediment reduction for each vegetation pattern, the runoff yield reduction effect of Pattern F was found to be the best, with a runoff yield reduction benefit of 19.65%. In terms of the sediment yield reduction benefit, Pattern B was the best, with a sediment yield reduction benefit of more than 70%. This is likely because the grass belt of Pattern F is near the top of the slope, and the amount of runoff yield and sediment yield from above is relatively small. In this case, the runoff yield and sediment yield from above are intercepted by the grass belt, so that the water from above is mostly used for infiltration and less runoff yield is formed. Therefore, the total runoff yield of Pattern F is small. However, as the grass belt of Pattern B is located at the junction of the upper slope and the lower slope, a large amount of runoff yield and sediment yield from above are intercepted through the grass belt, and the presence of sediment increases the slope roughness, which decreases the flow velocity and weakens the sediment carrying capacity of the runoff, and in turn, the total sediment yield is significantly reduced.

### Effects of different vegetation spatial patterns on sediment particle sorting

In this study, the change of sediment particles was mainly affected by slope runoff^27^. Issa et al^30^ found that runoff is one of the main factors for transporting sediment particles. As there is no vegetation cover on the upper slope of Pattern A, the slope runoff yield increased rapidly after the beginning of runoff production, and the transport capacity of slope runoff to coarse particles was strong, resulting in a high content of sand particles in eroded sediment (Fig. 7c). Therefore, the MWD of slope sediment particles was large. When the test reached a certain stage, rills gradually formed on the slope. At this time, the erosion sediment was composed of both inter rill erosion sediment and rill erosion sediment^54^. Due to the stronger erosion power of rill flow, runoff can carry more fine particles. Therefore, the content of fine particles in eroded sediment increased in the middle and later stage of the test (Fig. 7b), resulting in the weakening of the sorting of eroded sediment particles by runoff^55^.

Slope roughness was increased after vegetation was planted on the upper slope, which in turn altered the hydraulic characteristics of slope runoff, which reduced the runoff velocity and runoff erosion power of the slope and weakened the transport capacity of runoff to coarse particles. Therefore, the MWD of slope sediment particles was smaller than that of Pattern A. Different locations of grass belts on the upper slope had different effects on sediment particle sorting. Generally, under the hydraulic erosion condition of the slope with vegetation coverage, the erosion particles are mainly fine particles with high silt content and relatively little sand content, and as the distance of the vegetation from the top of the slope increases, silt of size 0.002–0.05 mm is more easily eroded (Table 5).

### Analysis on spatial optimization pattern of vegetation in the convex hillslope

Many studies show that vegetation has the dual function of both water storage and sediment reduction, and is therefore an effective method of soil and water conservation^54–56^. However, due to the limited water resources in the Loess Plateau, the overall capacity of vegetation in the area is limited. Excess vegetation leads to soil drying (forming a soil dry layer) and has an adverse impact on soil hydrological conditions^57^. Reasonable vegetation control structure can effectively improve soil properties and reduce or prevent water and soil loss, while unreasonable vegetation structure can lead to serious water and soil loss^58^. Therefore, optimizing the limited vegetation pattern of convex hillslopes and realizing the most effective regulation of soil and water loss are key factors in controlling soil and water loss. In the actual process of erosion and sediment yield, there is an optimal layout area of vegetation regulation erosion, that is, the optimal spatial pattern of vegetation. Vegetation is arranged in this area, and the vegetation can rely on the appropriate location, which can play the dual role of water and soil conservation.

Limited by the test conditions, the judgment coefficients of the vegetation relative position index and the fitting function of water storage and sediment yield reduction benefit in this study do not exceed 90%, resulting in a certain deviation between the calculated position and the actual situation. Therefore, when looking for the optimal area for vegetation regulation of erosion and sediment yield, it should be based on the principle of actual test conditions and supplemented by fitting function results. Based on the calculated results, it can be concluded that *Z* in the range of 1.11 to 1.17 is defined as the best vegetation spatial pattern. Combined with the actual situation, Pattern B is the best vegetation spatial pattern that ensures high runoff yield and reduced sediment yield.

## Conclusions

The laboratory experiments clearly showed that the different locations of grass strips placement have a significant effect on both runoff and sediment on convex hillslope. After the vegetation was deployed on the upslope, the total runoff yield was reduced by 1.79–19.65%, and the total sediment yield was reduced by 21.19–70.22%, and the eroded particles were mainly fine particles dominated. Under different vegetation spatial patterns, the effect of vegetation cover on reducing sediment yield was greater than that of reducing runoff yield. When the grass strip was planted near the lowermost point of the upslope, and it could reduce total runoff yield and total sediment yield by 12.13% and 70.22%, respectively. And based on the results of the calculation of the vegetation relative position index, it was determined that Pattern B is the only vegetation position that can assure both high runoff yield and sediment yield reductions. In the process of erosion control in the loess hill and gully area in the future, vegetation can be planted on the lower part of the upslope, so as to better play the function of vegetation to reduce runoff yield and sediment yield.

## Data Availability

The datasets generated and analysed during the current study are not publicly available due this experiment was a collaborative effort, the trial data does not belong to me alone but are available from the corresponding author on reasonable request.
